# Gastroesophageal Reflux in Children with Asthma

**DOI:** 10.3390/children9030336

**Published:** 2022-03-02

**Authors:** Vasile Valeriu Lupu, Ingrith Miron, Elena Tarca, Laura Mihaela Trandafir, Dana-Teodora Anton-Paduraru, Stefana Maria Moisa, Magdalena Starcea, Andrei Cernomaz, Lucian Miron, Ancuta Lupu

**Affiliations:** 1 Pediatrics Department, “Grigore T. Popa” University of Medicine and Pharmacy, 700115 Iasi, Romania; valeriulupu@yahoo.com (V.V.L.); ingridmiron@hotmail.com (I.M.); trandafirlaura@yahoo.com (L.M.T.); antondana66@yahoo.com (D.-T.A.-P.); magdabirm@yahoo.com (M.S.); anca_ign@yahoo.com (A.L.); 2 Department of Surgery II—Pediatric Surgery, “Grigore T. Popa” University of Medicine and Pharmacy, 700115 Iasi, Romania; 3 3rd Medical Department, “Grigore T. Popa” University of Medicine and Pharmacy, 700115 Iasi, Romania; lucmir@gmail.com

**Keywords:** gastroesophageal reflux, asthma, 24 h esophageal pH-metry, Boix-Ochoa score, children

## Abstract

Background: Nowadays it is considered that a specific causal relationship exists between asthma and gastroesophageal reflux (GER), because of the aspiration of gastric refluate which leads to and maintain spasticity even real crisis of paroxystic expiratory dyspnea. This study explores this relationship and evaluates the results after treatment. Methods: 56 children diagnosed with asthma, hospitalized in a regional center of pediatric gastroenterology in Northeast Romania, underwent 24-hour continuous esophageal pH monitoring in order to establish the presence of GER. The Boix-Ochoa score was used to interpret the results. Proton pump inhibitors were administered to those with GER and the patients were reevaluated after 2 months. Results: 39 patients (69.64%) had GER, with a Boix-Ochoa score above normal (N < 11.99), and 17 patients (30.36%) had normal score. After administering proton pump inhibitors for 2 months, 7 patients still had high Boix-Ochoa score (17.95%). The result of this analysis shows that the presence of asthma increases the chance of GER by 2.86 times. Conclusions: In children with asthma we have to look for GER in order to treat, because it can help the treatment of asthma or even solve some cases resistant to standard treatment.

## 1. Introduction

Epidemiological studies have shown that there is a close connection between gastroesophageal reflux and asthma, especially in children [1,2]. The American College of Gastroenterology guidelines define gastroesophageal reflux disease (GERD) as “symptoms or mucosal damage produced by the abnormal reflux of gastric contents into the esophagus” [3].

Asthma is a chronic disease of the airways which is characterized by obstructed airflow, bronchial hyperactivity, and inflammation [4]. Different comorbidities can accentuate symptoms or favor the emergence of asthma. In a study that investigated the prevalence of symptomatic gastroesophageal reflux (GER) in asthmatics based on a questionnaire, 77% of the 109 participants in the study suffered from epigastralgia, and 55% experienced regurgitation. Symptoms were more pronounced than in the control group [5]. GER can present with a variety of symptoms, ranging from digestive to respiratory and neurobehavioral [6].

Gastroesophageal reflux (GER) has been recognized as a triggering factor in asthma and its medical management is recommended [7]. The improvement of respiratory symptoms through medical therapy or anti-reflux surgery further confirms the interrelation between GER and respiratory symptoms—especially asthma-like manifestations [8]. The association of GER with bronchial asthma is considered a special causality relationship in which the continuous aspiration of gastric refluate generates the development and maintenance of spasticity and the real crises of paroxysmal expiratory dyspnea. Although these two diseases often emerge together, the precise relationship between GER and asthma remains unclear. This study aims to investigate this relationship and evaluate the results of the appropriate treatment.

## 2. Materials and Methods

A retrospective study was performed on 56 children, between 3 and 18 years old diagnosed with bronchial asthma, hospitalized in a regional center of pediatric gastroenterology in Northeast Romania, for a period of 3 years. The criteria for their inclusion in the study were the presence of bronchial asthma symptoms (morning dysphonia, persistent night cough, night and effort-related wheezing, laryngospasm, apnea), resistance to standard treatment, bronchial asthma with atypical history, and non-allergic bronchial asthma with late debut (in adolescents). Diagnoses were established based on anamnesis, objective examination, and paraclinical examinations. Patients’ biological samples and medical records were considered, and the data were completed—to the extent to which they were available—by patients’ hospital discharge certificates from the pneumology clinic.

Informed consent was obtained from all patients/caregivers, and the “St. Mary” Children Emergency Hospital Ethics Committee’s approval was obtained for publishing this study.

The patients were evaluated for the presence of GER by continuous monitoring during 24 h of the esophageal pH. The Boix-Ochoa score was used to interpret the results as recommended by literature for pediatric age (N < 11.99). This test differentiates the physiological from pathological reflux [9]. We used as device the Medtronic Digitrapper pH 100, SN 37660, with Polygram Net TM pH Testing Application and Kinetics 24 and multi-use catheters ComforTec by Sandhill. The pH-metry is based on the idea that the progression of the acid gastric content in the esophagus during reflux causes a decrease in intraesophageal pH. The method involves measuring the pH in the lower part of the esophagus for a certain period (in our study 24 h) by using an electrode located 5 cm above the cardia and attached to a portable pH-meter with batteries [10,11,12,13,14].

The equipment must be calibrated before each use in 2 fluids with pH 1 and 7. Prior to the procedure, the child should not consume drink and food for at least 6 h for those over 1 year of age, and for at least 3 h for infants. [15]. Antacid treatment should be interrupted at least six hours in advance, anti-H2 receptor therapy three days in advance, proton pump inhibitor therapy seven days in advance, and prokinetic therapy 48 h in advance [16]. The child is placed in the left lateral decubitus (for infants and children) or in a seated position (for children over the age of 5–6), while the examiner stands to the right side of the patient. The electrode is lubricated first, then inserted nasally up to 5 cm above the cardia. Each child or relative is instructed to record any symptoms, time and position of the body (supine position, standing) and at the same time to press the button on the device [11,12,13,14]. The test is positive if the pH decreased below four for more than five seconds [17]. All children with a positive score received treatment with proton pump inhibitors (1 mg/kg/day) and were reevaluated after 2 months of treatment.

We used SPSS 20 for statistical data processing. Pearson parametric correlation was used for the correlation analysis and the correlation coefficients were calculated for a confidence interval (CI) of 95%. Logistic regression offers a useful means for the modeling of the dependence of a dichotomous response variable on one or several explanatory variables called “predictors”, which can be categorical or continuous. Risk is mathematically modeled as a predictor variable in the form of an equation.

## 3. Results

**Figure 1:**
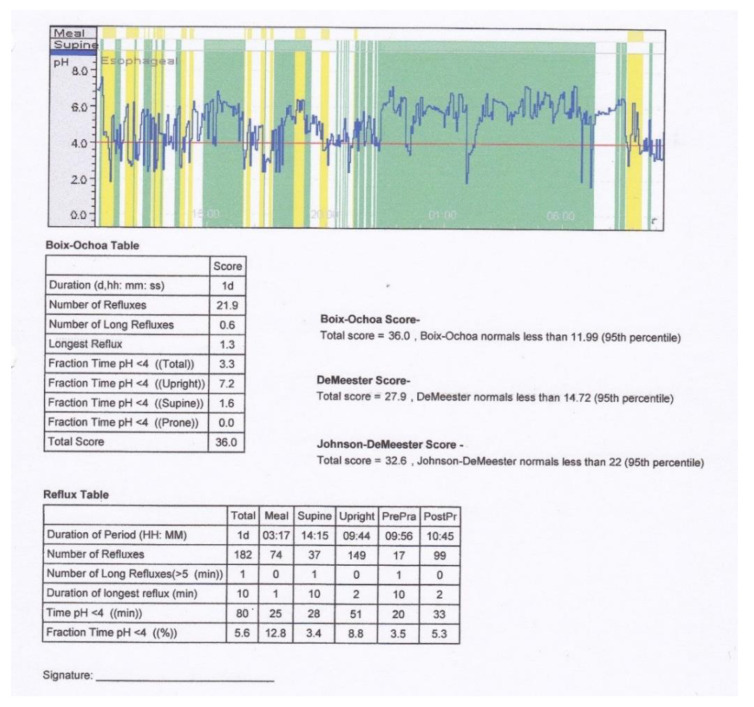
Positive pH-metry (from the clinic’s collection).

**Figure 2:**
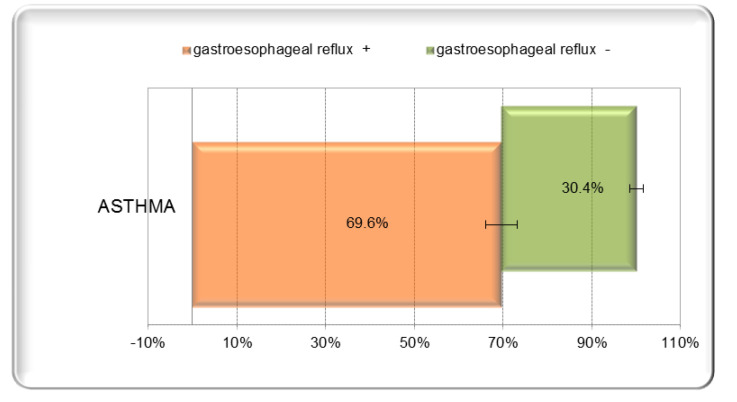
The association of GER with asthma.

According to the data in the specialized literature, we considered as reflux any positive Boix-Ochoa score (>11.99) [15] (Figure 1). A total of 39 (69.64%) of the 56 children with bronchial asthma under study, of both sexes and from both rural and urban areas, had GER, as demonstrated by a positive Boix-Ochoa score, while 17 children (30.36%) had a negative score (Figure 2). The result of the statistical analysis demonstrates the fact that the presence of asthma increases the chances of GER by a factor of 2.86 (Table 1). Regarding age, we divided the children into two groups (3–9 years and 10–18 years, respectively) (Table 2).

All 39 children with bronchial asthma and gastroesophageal reflux (demonstrated by a positive Boix-Ochoa score at pH-metry) were submitted to specific treatment with proton pump inhibitors (PPI), besides the general measurements specific to this disease. Accordingly, postural therapy and diet measures specific to their age were applied to all children. The medication administered consisted of PPI-Omeprazole or Esomeprazole. The usual doses were administered daily for 2 months. After two months of treatment, the pH was measured again. In 32 patients, the pH-metric values became normal, while the Boix-Ochoa score was still positive in seven patients (17.95%). Clinical improvement was obvious from the first weeks of treatment in those patients with favorable evolution.

## 4. Discussion

Several extra-esophageal manifestations of GERD have been described in both adults and children. While, for a part of these, a causal relationship has been proven, for others the association is casual [18]. It is important to investigate the presence of GER in patients with asthma who are resistant to classical treatment. Testing for GER in all patients with asthma has also been recommended [19]. Most people with asthma have symptoms specific to GER, but 24% do not [20]. GER can induce bronchoconstriction by two mechanisms: the direct mechanism (the reflux theory), by which the pulmonary tree comes into contact with the gastric refluate, and the indirect mechanism (the reflex theory), by which the gastric acid can stimulate the vagal nerve terminations in the lower part of the esophageal wall [21]. Twenty-four hour esophageal pH-metry is the most widely used method for diagnosing GER. The sensitivity of pH-metry is higher than 85% and its specificity is 95% [22].

We didn’t encounter side effects of pH-metry in small children, besides nose bleeding in some cases when we introduced the naso-esophageal tube, which stopped spontaneously. It is known that introducing the tube does not conform to the usual habits of children, especially younger ones.

The author of a study regarding pediatric laryngopharyngeal reflux which used the oropharyngeal pH-Dx system for pH-monitoring reported that there were few cases in which there was some resistance in probe insertion associated with mild epistaxis; out of 26 children, 4 children did not complete the 24 h recording because 3 children were able to pull the probe out, while there was 1 case where the probe was displaced in the nasopharynx [23].

The limitations of this study are that pH-metry cannot detect low-acid and nonacid reflux episodes. The severity of pathologic acid reflux does not consistently correlate with symptom severity or demonstrable complications. Furthermore, the data reflect a single clinical center with a low prevalence of asthma. Moreover, due to the retrospective design, some patients were lost along the way, as they failed to present for re-evaluation.

GER was present in 69.64% of all patients with asthma. This result is in accordance with other studies, which reported a frequency of 65–66% [19,22]. The association of GER with bronchial asthma is well proven, both in children and in adults. Thus, in a study conducted on 31 adults, 17 patients had a positive pH-metry score [24]. Although the use of proton-pump inhibitors is the treatment of choice for GERD, about a third of patients do not respond to this therapy [25], which can explain why 17.95% had a positive Boix-Ochoa score after 2 months of PPI treatment. In one study, children with persistent moderate asthma and reflux who received anti-reflux treatment including PPI used significantly less medication to control their asthma [26].

Empiric therapy with PPIs was considered the initial diagnostic step in children suspected of having GERD-related symptoms. People who have asthma and are unresponsive to PPI, without overt regurgitation, usually have either no reflux or causes other than GERD. In this group, PPI treatment should be discontinued. In those with GERD as a contributing factor, acid suppressive therapy should be continued and other etiologies requiring concomitant treatment should be optimally treated [27]. Children with respiratory complications, including asthma, are considered most likely to benefit from anti-reflux surgery when medical therapy fails, but additional studies are needed to confirm this assumption [28]. A limitation of the study consists of the fact that the causality between asthma and GER is difficult to establish, as each of these conditions can induce the other, which can lead to interpretation errors. Asthma can provoke reflux by creating negative intrathoracic pressure, which is why the gastric content exceeds the lower esophageal sphincter. In addition, the medication used to treat bronchial asthma (bronchodilators) can aggravate reflux [29].

There are studies which demonstrate that PPIs are associated with a significant risk of acute interstitial nephritis, chronic kidney disease, and kidney disease progression; a higher risk of incident dementia, and also an increased risk of community-acquired pneumonia and hospital-associated pneumonia [30,31,32,33]. The use of PPIs is furthermore associated with cardiovascular events and an increased risk of death [33,34].

The authors of some studies report that treatment with a proton-pump inhibitor (PPI) in children with asthma was not only unsuccessful, but that adverse effects were common, including an increased prevalence of symptomatic respiratory infections. The presence of GERD is very common in children with asthma, but approximately half of the children are asymptomatic. The Food and Drug Administration-approved doses of proton-pump inhibitors did not improve the asthma evolution in children with asymptomatic GERD on school-age children with poorly controlled asthma by inhaled corticosteroids; this is the reason why the same authors concluded that GERD and asthma can only be associated by chance, because this is very prevalent in the general population [35,36].

On the other hand, in adults, aggressive acid suppressive treatment with proton-pump inhibitors has been suggested to ameliorate asthma evolution in asthmatics with GERD. A study of 30 nonsmoking adult asthmatics with GERD reported that a three-month regimen of proton-pump inhibitors improved asthma symptoms and also pulmonary function in 73% of the patients [37]. The authors of a study investigated the efficacy of proton-pump inhibitor on GERD linked chronic cough and they found that proton-pump inhibitor therapy relieves GERD linked chronic cough, but it was suggested a double-standard dose of the proton-pump inhibitor for 2 to 3 months [29].

For correct diagnosis, it is important to differentiate asthma from GERD. A patient’s medical history should be evaluated carefully, including atopy or allergic rhinitis, affected children have eosinophilia and a high level of Ig E. If children have eosinophilia associated with digestive symptoms, parasitic disease should be excluded [38]. A negative stool test and a parasite antibody test must be obtained. Eosinophilic gastroenteritis could present with eosinophilia or normal upper gastrointestinal endoscopy in people who have asthma [39,40]. For diagnostic certainty, other specific laboratory tests should be conducted (lung function tests, skin prick test, and FENO analysis). It is important to combine different methods to make a correct diagnosis.

## 5. Conclusions

Asthma is a strong reason for the evaluation of the presence of gastroesophageal reflux by using continuous esophageal pH-metry for 24 h, especially when there is a weak response to asthma therapy. The bronchial spasm triggered and maintained by aspirating the refluxed acid remains the most believable explanation for this relationship and association. The statistical test results demonstrated that the presence of asthma increases the chances of GER by 2.86. Proper therapy of GER resolves or at least help in the treatment of asthma.

## Figures and Tables

**Table 1:** Coefficients and Wald tests for logistic regression.

	β	S.E.	Wald	Sig.	Exp(β) OR	95% C.I. for EXP (B)	
						Lower	Upper
ASTHMA	0.769	0.354	4.722	0.019	2.863	1.632	3.927

χ^2^ Statistical test = 0.556 (goodness of fit based on deciles of risk); degrees of freedom = 6; *p* = 0.997; 95% C.I.

**Table 2:** Distribution of cases by age.

	Children with Asthma		Children with GERD and Asthma		Children with Asthma, without GERD	
3–9 years	42	33 boys	28	23 boys	14	10 boys
		9 girls		5 girls		4 girls
10–18 years	14	8 boys	11	6 boys	3	2 boys
		6 girls		5 girls		1 girl
Total	56		39		17	

## Data Availability

The data that support the findings of this study are available from the corresponding author upon reasonable request.
